# A bicentric study on the prevalence and clinical relevance of subarachnoid hyperdensities on flat-detector CT after thrombectomy in dominant, co-dominant, and non-dominant M2 occlusions

**DOI:** 10.1007/s00234-025-03679-x

**Published:** 2025-06-19

**Authors:** Mousa Zidan, Bettina Lara Serrallach, Mattia Branca, Felix Bode, Eike Piechowiak, Thomas Meinel, Nils Christian Lehnen, Tomas Dobrocky, Johannes Kaesmacher, Franziska Dorn

**Affiliations:** 1 Department of Neuroradiology, Bonn University Hospital, Bonn, Germany; 2 Department of Diagnostic and Interventional Neuroradiology, University Hospital Bern, Bern, Switzerland; 3 Department of Clinical Research, CTU Bern, University of Bern, Bern, Switzerland; 4 Department of Vascular Neurology, Bonn University Hospital, Bonn, Germany; 5 Department of Neurology, University Hospital Bern, Bern, Switzerland; 6 Department of Neuroradiology, LMU-Klinikum der Universität München Medizinische Klinik und Poliklinik IV, Munich, Germany

**Keywords:** MVO, FDCT, Mechanical thrombectomy, Co-dominant, Non-dominant M2 occlusions

## Abstract

**Background:**

Subarachnoid hyperdensities (SH) on flat-detector CT (FDCT) after mechanical thrombectomy (MT) are associated with less favorable clinical outcomes. We aimed to further elucidate the prevalence and clinical significance of SH following MT, especially in patients with dominant, co- and non-dominant M2 occlusions.

**Methods:**

728 patients from two comprehensive stroke centers were assessed for the presence of SH on FDCT. The primary outcome was the presence of SH on FDCT. The secondary outcome was modified Rankin Scale scores (mRS) at 90 days. Baseline procedural characteristics and clinical outcomes were analyzed using group comparisons and multivariable logistic regression. To remove the effect of confounding factors, a logistic regression model was built using inverse probability weighting.

**Results:**

In total, 411 patients were included. Prevalence of SH on FDCT was 171/411 (41.6 %), with particularly high prevalence in co- and non-dominant M2 occlusions (63%) and dominant M2 occlusions (53.9%). The occurrence of SH was independently associated with poor functional outcomes (adjusted OR for mRS at 90 days: 1.5; 95% CI, 1.1–2.2) and increased mortality (aOR: 1.7; 95% CI, 1.0–2.8). Increased risk of developing SH was particularly evident in patients with co- and non-dominant M2 occlusions (*P* < 0.001 OR = 3.78; 95% CI, 2.18–6.57) and dominant M2 occlusions (*P* < 0.001 OR = 3.07; 95% CI, 1.68–5.59) compared to large vessel occlusions. A higher number of device passes, specifically between 3 and 6 and more than 6, show an effect on the occurrence of SH *P* < 0.001 OR = 2.75; 95% CI, 1.56–4.84 and P = 0.02 OR = 3.45; 95% CI, 1.17–10.16 compared to fewer passes (1–3).

**Conclusion:**

SH are common after MT, especially in M2 occlusions. They are associated with poorer functional outcomes in patients with co- and non-dominant M2 occlusions and higher numbers of device passes (>3).

**Supplementary Information:**

The online version contains supplementary material available at 10.1007/s00234-025-03679-x.

## Introduction

The results of studies on periprocedural subarachnoid hyperdensities (SH) after mechanical thrombectomy (MT) have been inconsistent. Initially, SH were described as a rare and benign finding [1, 2]. However, more recent studies have suggested that SH are more frequent and associated with neurological deterioration and increased mortality [3, 4]. They were found to be more common in patients with distal occlusions and following multiple device passes.

Several mechanisms for the development of SH have been discussed, the most obvious being mechanical disruption of endothelial integrity during MT, for example avulsion of perforators caused by instruments such as stent retrievers (SR) and guidewires [5]. Navigating and maneuvering the SR through smaller-diameter, thinner-walled vessels, such as M2 branches, causes more traction force, resulting in stretching and rupture of the small perforating arteries [6]. In addition, the presence of a genu at the level of the transition from the M1 to the M2 segment hinders the smooth retraction of the SR [7].

The difficulties of gaining access to the occluded vessel due to its tortuous anatomy and narrow caliber may lead to higher rates of SH in patients with medium vessel occlusion (MVO) compared to those with large vessel occlusion (LVO). Despite the lack of clear guideline-based recommendations, MT is considered increasingly more often for patients with relevant neurological deficits due to a MVO.

Outcomes and complication rates after MT have been found to be similar in patients with M2 and M1 occlusions [8], including dominant and non-dominant M2 occlusions [9]. Conversely, a multicentric investigation reported twice as many complications in patients with MVOs compared to LVOs [10]. Higher rates of intracranial hemorrhage, but with higher rates of functional independence overall, following M2 occlusions have also been reported [11]. This discrepancy can be attributed to wide variations of the branching pattern of the middle cerebral artery (MCA) [12, 13] and consequently of the arterial territory at risk.

Some neurointerventional centers acquire post-interventional flat-detector computed tomography (FDCT) to rule out complications. FDCT can detect findings that are not visible during conventional digital subtraction angiography (DSA).

In this bicentric investigation, we aimed to assess the prevalence and clinical significance of SH on FDCT following MT, especially in patients with dominant, co- and non-dominant M2 occlusions, and to identify risk factors and predictors.

## Materials and methods

In this retrospective study, anonymized data were analyzed after ethical review board approval, and informed consent was waived. All patient data and procedures conducted in this study complied with the guidelines of the Declaration of Helsinki.

### Study sample

**Fig. 1:**
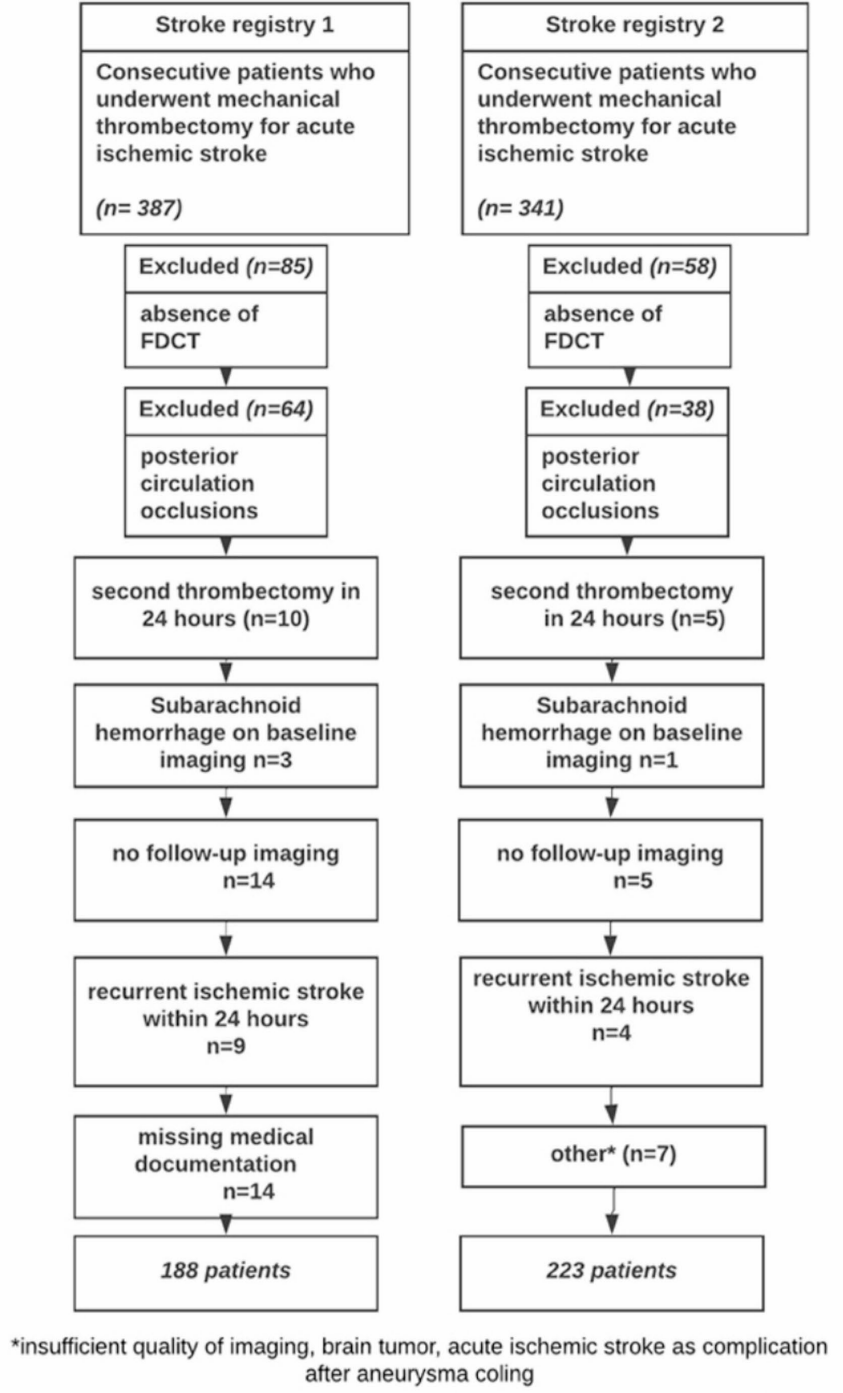
Patient flowchart with inclusion and exclusion criteria

Patients with acute ischemic stroke who underwent MT between January 2020 and December 2022 in two tertiary stroke centers in Germany and Switzerland were consecutively analyzed. Inclusion criteria were (a) patients with anterior circulation vessel occlusion; (b) patients received post-interventional FDCT. Exclusion criteria were (a) intracranial hemorrhage at baseline imaging; (b) recurrent ischemic stroke within few days; (c) no available follow-up imaging; (d) lack of clinical documentation in the medical records. (Fig. 1) shows the patient flowchart with the inclusion and exclusion criteria.

### Study end points and outcome measures

The primary outcome was the presence of SH on FDCT, especially in medium vessel occlusions. The secondary end point was modified Rankin Scale (mRS) at 90 days. National Institutes of Health Stroke Scale (NIHSS) score and mRS at admission, discharge and at 90 days were evaluated by an experienced neurologist.

### Endovascular recanalizing procedures

MT was performed under general anesthesia via a transfemoral approach. Materials were selected based on the judgment and experience of the treating physician. There were no constraints regarding the use of SR or aspiration maneuvers. The first-line technique in both tertiary centers is combined stent retriever and distal aspiration thrombectomy. This was the routine approach in most cases, although device and strategy selection ultimately remained at the discretion of the treating neurointerventionalist. As a result, different SRs with different length and diameter have been used (including but not limited to e.g. Catch mini for distal occlusions). Furthermore, the choice of technique was influenced in some cases by participation in ongoing clinical trials during the inclusion period. For instance, once center contributed cases to the SWIFT Direct study and both centers contributed to the DISTAL and ESCAPE-MeVO trials.

In patients with tandem occlusions, a retrograde approach (intracranial first) was preferred and an antegrade approach was chosen only if the passage of the proximal occlusion was otherwise impossible. Acute carotid artery stenting was performed when indicated. If no intravenous lysis was given, patients initially received intravenous heparin, 3000 UI, and an additional 1000 IU for every additional hour’s duration of the intervention. Patients needing extracranial stents received intravenous acetylsalicylic acid (ASA) 500 mg before stent implantation. This was followed by dual antiplatelet therapy (DAPT) within 24 h after the intervention, and therefore always after the follow-up CT or MRI. DAPT consisted of ASA and Clopidogrel or ASA and Ticagrelor. Platelet function test was performed to identify partial/non-responders.

Eligibility for intravenous tissue plasminogen activator IV (tPA) was evaluated, and tPA treatment was administered according to the guideline’s recommendations.

### Image acquisition

#### FDCT

The following post-interventional images were acquired in the angio-suite:

- FDCT (Allura Xper FD 20 flat-detector with Xper-CT, Philips, Best, The Netherlands). The acquisition parameters have been reported in detail elsewhere [14].
- The Sine Spin FDCT (ARTIS icono biplane; Siemens, Germany). The acquisition parameters have been previously reported [15].

#### Follow-up imaging

One center obtained all follow-up scans with an IQon spectral scanner (Philips Healthcare, Best, The Netherlands). Acquisition parameters have been reported in detail elsewhere [16]. Spectral postprocessing was performed using dedicated software (IntelliSpace Portal, Philips Healthcare, The Netherlands). A virtual non-contrast (VNC) scan, iodine-removed map, and an iodine map were generated.

The other center obtained follow-up CT with a Somatom Definition Edge scanner (Siemens, Erlangen, Germany). Follow-up MRI studies were performed at a magnetic field strength of 1.5 T or 3 T. Follow-up protocols mostly included axial DWI and a matching ADC map, axial FLAIR, 3D TOF-MRA of the intracranial arteries, and axial SWI.

### Image analysis

#### Post-interventional FDCT

**Fig. 2:**
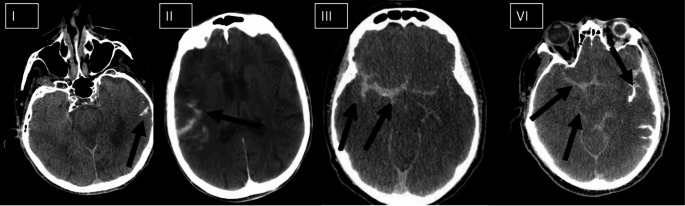
Grade I: SH in 1 or 2 adjacent sulci; grade II, SH in ≥ 3 adjacent sulci but confined to a single lobe; grade III, diffuse sulcal SH affecting ≥ 2 lobes; and grade IV, confluent sulcal hyperdensities affecting ≥ 2 lobes and extension into basal cisterns

The presence of SH on FDCT was documented using a visual grading scale (I–IV) as described previously [4]. Grading was as follows:

- Grade I: SH in 1 or 2 adjacent sulci.
- Grade II: SH in ≥ 3 adjacent sulci but confined to a single lobe.
- Grade III: diffuse sulcal SH affecting ≥ 2 lobes.
- Grade IV: diffuse sulcal hyperdensities affecting ≥ 2 lobes and intraventricular extension or extension into basal cisterns.

(Fig. 2) depicts the visual grading scale of SH.

#### Analysis of blood–brain barrier disruption and hemorrhage

VNC scans and iodine-only images were used to classify detected SH on follow-up dual-energy (DE)-CT as follows:

1. Hemorrhage: when associated with hyperdense correlate on the VNC images.
2. Exudate of contrast medium into extracellular spaces when not associated with a hyperdense correlate in the VNC scan but only in iodine-only images.

In the center, where the primary follow-up imaging was MRI. The presence of subarachnoid hemorrhage (SAH) was established by the presence of a hypointense lesion in the subarachnoid space on gradient recalled-echo (GRE) SWI with a correlating hyperintense signal on the FLAIR sequence or the persistence of a preexisting hyperdense lesion from FDCT on follow-up CT. Otherwise, the appearance of a new hyperdense lesion within the subarachnoid space on the immediate post-intervention CT scan was classified as either pure SAH or a combination of SAH and contrast extravasation. Pure SAH was identified as a hyperdense lesion with maximum Hounsfield units (HU) of < 90, while a lesion with maximum HU of ≥ 90 was classified as a mixture of SAH and contrast extravasation as previously described [1].

Hemorrhagic transformation was classified either as hemorrhagic infarct or parenchymal hematoma according to the European Cooperative Acute Stroke Study (ECASS) classification.

#### Definition of MCA branches and assignment of M2-dominance

**Fig. 3:**
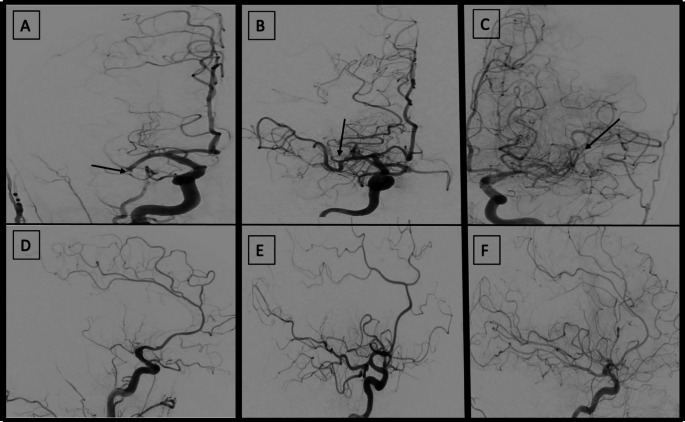
(**A**) Case example of an AP view showing a dominant caliber M2 occlusion on DSA. (**B**) Case example of an AP view of a co-dominant caliber M2 occlusion on DSA. (**C**) Case example of an AP view of a non-dominant caliber M2 occlusion on DSA. Arrows indicate occlusion sites. (**D, E, F**) Corresponding lateral views on DSA pre-MT demonstrating the associated perfusion deficit

M1 refers to the segment that runs horizontally along the sphenoid wing up to the limen insulae, where it curves postero-superiorly (genu) into the insula. From the genu, M2 branches run vertically, M3 branches spread laterally and exteriorly from the Sylvian fissure, and M4 branches run along the outer surface of the brain’s convexity. In up to 4/5 individuals the MCA typically divides into a bifurcation [12]. These trunks are in hemodynamic balance and vary considerably in the dominance of one trunk over the other [13]. There is no consensus in the literature on how to assign dominance to different M2 branches, and it has been classified arbitrarily in previous studies. We utilized both DSA and CTA images to decide on caliber dominance. We classified branches that had a larger diameter than others or were associated with a large perfusion deficit, > 50% MCA territory, as dominant. Co-dominance was assigned to branches that supported up to 50% of the MCA territory. A smaller-caliber branch supplying < 50% of MCA territory when paired with other clearly dominant or co-dominant branches, was considered non-dominant. This method was previously implemented in a retrospective analysis of the MR CLEAN registry [9]. Classification of the M2 branches was assessed by an experienced neuroradiologist and a board-certified radiologist. Examples of M2 caliber dominance as assigned in the DSA images, are provided in (Fig. 3).

#### Statistical analysis

We collected data on demographic characteristics, vascular risk factors, premedication, NIHSS score, mRS score at admission, at discharge, and at 90 days, site of occlusion, Alberta stroke program early CT score (ASPECTS), number of maneuvers, and final modified treatment in cerebral infarction (mTICI) score. Distal intracranial ICA and M1 occlusions were defined as LVO; M2, A1, and A2, as MVO; and M3 and A3, as distal vessel occlusion.

Various clinical and radiological variables were analyzed to establish their significance in the presence of SH following MT. Data distribution was tested using the Shapiro-Wilk test. We performed chi-squared tests for categorical variables and two-sided t-tests or Wilcoxon-Mann-Whitney U for continuous variables. Descriptive statistics are presented as frequencies and percentages for categoric variables and median with interquartile range for continuous variables.

Multivariate logistic and ordinal logistic regression analysis were used to examine the role of selected factors in the occurrence of SH (age, sex, NIHSS on admission, intravenous thrombolysis, number of device passes, previous anticoagulant and antiplatelet medication, and site of occlusion).

In addition, multiple regression analysis was used to assess the influence of MVOs and number of device passes on the occurrence of SH. For initial classification, M2 branches were categorized as dominant, co-dominant, or non-dominant based on caliber and perfusion territory. For statistical analysis, due to the small number of non-dominant cases, we combined co-dominant and non-dominant occlusions into one group to ensure adequate power and interpretability. The analysis was performed on dominant, co- and non-dominant M2 occlusions and the number of device passes was grouped into three categories (1–3, 4–6 and > 6) to recognize possible thresholds.

To examine the effect of removing confounding factors, a logistic regression model was built to estimate the probability of exposure using inverse probability weighting. To this end, weighted analysis was applied to the following variables: age, sex, site of occlusion, IVT with tPA, prestroke mRS, ASPECTS, and NIHSS. These are considered the most reliable predictors for functional outcome after stroke [17]. Subsequently, the effect of different SH categories (0 to IV) on outcomes of interest such as the mRS score at 90 days and mortality at 3 months (equivalent to mRS 6) was modeled. Results are presented as adjusted ORs (aORs) with 95% CIs for the different outcomes. We provided p values and adjusted it with Benjamini-Hochberg method to account for type I error occurring from multiple testing among the groups.

A statistically significant difference was defined as *P* < 0.05. All statistical analyses were performed with R statistical and computing software, R 4.3.1 (http://www.r-project.org/) and/or Stata 17.0 (StataCorp). Although formal power calculations were not possible due to the retrospective design of the study, the sample size was deemed sufficient to provide meaningful effect size estimates and to allow for a thorough evaluation of the study objectives.

## Results

Seven hundred and twenty-eight patients from the two stroke registries, who underwent MT between January 2020 and December 2022 were analyzed. After applying the inclusion and exclusion criteria, we included 411 patients. The most common reasons for exclusion were the absence of FDCT after MT (*n* = 143) and posterior circulation occlusions (*n* = 102). Among 234 patients with LVOs, 185 had M1 and 49 had ICA occlusions. MVOs were observed in 159 patients with M2 involvement. SVOs included 16 patients with M3 and two patients with A3 segment occlusions.

### Prevalence of SH

Of the 411 patients, 171 had SH on FDCT (41.6%); 77 were classified as SH I (45.0%), 54 as SH II (31.6%), 28 as SH III (16.4%) and 12 as SH IV (7.0%). Follow-up imaging showed SH in 27.7% of all patients (114/411) and in 66.6% of those who had initially had SH (114/171); SH I 75.4% (86/114), SH II 17.5% (20/114) and SH III 7.0% (8/114).

The incidence of active extravasation seen on DSA was greater at higher SH grades (SH I, 0/77 (0%); SH II, 1/54 (1.9%); SH III, 4/28 (14.3%); SH IV, 8/12 (66.7%)).

Baseline characteristics, procedural characteristics and a comparison of outcome measures between patients with no SH on FDCT and patients with SH grades (I–IV) are summarized in (Table 1).

**Table 1:** Baseline and procedural characteristics stratified by subarachnoid hyperdensities seen on flat-panel detector computer tomography

Group	Total	SH 0	SH I–IV	*P*
Median (lq-uq), [*N*], % (*n*/*N*)	*411*	*240*	*171*	
Age at stroke (years)				
	77 [64.6–85.0]	78 [67.0–86.0]	75.1 [62.7–83]	0.21
Sex				
Male	48% (199)	51% (123)	44% (76)	0.21
Female	52% (212)	49% (117)	56% (95)	
Baseline comorbidities:				
Hypertension	76% (313)	77% (185)	75% (128)	0.69
Medical history of diabetes	23% (94)	22% (54)	23% (40)	0.93
Smoking	22% (92)	23% (56)	21% (36)	0.64
Hyperlipidemia	64% (262)	63% (152)	64% (110)	0.92
Atrial fibrillation	41% (167)	44% (106)	36% (61)	0.1
Initial ASPECTS NIHSS on admission	13 [7.5–19.0]	8 [6.0–9.0] 14 [8.0–19.0]	8 [6.0–9.0] 13 [6.0–19.0]	0.93 0.23
Prestroke mRS	0 [0.0–2.0]	0 [0.0–2.0]	0 [0.0–2.0]	>0.99
Baseline intracranial occlusion site				
LVO	57% (234)	66% (158)	44% (76)	< 0.001**
MVO	39% (159)	30% (73)	50% (86)	
SVO	4.4% (18)	3.8% (9)	5.3% (9)	
Number of passes				
Intravenous thrombolysis	2 [1.0–3.0] 44% (182)	1 [1.0–2.0] 45% (107)	2 [1.0–4.0] 44% (75)	< 0.001** 0.96
mTICI score				
0	3.4% (14)	1.2% (3)	6.4% (11)	< 0.001**
1	0.97% (4)	0.42% (1)	1.8% (3)	
2a	2.4% (10)	2.5% (6)	2.3% (4)	
2b	49% (202)	45% (107)	56% (95)	
3	44% (181)	51% (123)	34% (58)	
mRS at 3 months				
	3 [1.8–6.0]	3 [1.0–5.0]	3 [2.0–6.0]	0.33
NIHSS at 24 h (or DC)				
	6 [2.0–13.8]	6 [2.0–13.0]	6 [2.0–14.0]	0.49

*NIHSS* National Institutes of Health Stroke Scale, *ASPECTS* Alberta Stroke Program Early CT Score, *mRS* modified Rankin scale, *LVO* large vessel occlusion, *MVO* medium vessel occlusion, *SVO* small vessel occlusion, *mTICIs* modified treatment in cerebral infarction score. * Statistical significance, *P* < 0.05 and values are marked in bold. ** Statistical significance, *P* < 0.001 and are marked in bold

The baseline intracranial occlusion site of the SH IV group was characterized by a higher proportion of LVO (8/12) than in any other SH group. Additional baseline characteristics stratified by SH group are shown in (supplemental material 1).

### Functional outcome and mortality

The adjusted analysis using inverse probability weighting showed unfavorable clinical outcomes in patients with SH (aOR = 1.5; 95% CI, 1.1–2.2). The effect was most pronounced when comparing patients with moderate SH (SH II) to patients with no SH and when comparing patients with SH grades II–IV to patients with minimal or no SH. Patients with SH had increased odds for higher mRS at 90 days (aOR = 2.4; 95% CI, 1.2–5.0; aOR = 2.1; 95% CI, 1.2–3.6), respectively. The odds of higher mortality in the SH groups II–IV were also increased compared with patients with minimal (SH I) or no SH (aOR = 1.7; 95% CI, 1.0–2.8). The results of the adjusted group analysis are provided in (Table 2).

**Table 2:** Adjusted results for the outcomes and group comparisons

Outcome	aOR (95%-CI)	*P* value	Adjusted *P* value
mRS at 90 days			
SH 0 vs. SH I-IV	1.5 (1.1 to 2.2)	0.02*	
SH 0 vs. SH I	1.3 (0.7 to 2.5)	0.31	0.42
SH 0 vs. SH II	2.4 (1.2 to 5.0)	0.01*	0.04*
SH 0 vs. SH III	3.1 (1.1 to 8.6)	0.02*	0.05
SH 0 vs. SH IV	0.7 (0.2 to 2.7)	0.61	0.61
SH 0-I vs. SH II-IV	2.1 (1.2 to 3.6)	0.01*	
Mortality at 90 days			
SH 0 vs. SH I-IV	1.7 (1.1 to 2.8)	0.02*	
SH 0 vs. SH I	1.1 (0.9 to 1.2)	0.21	0.28
SH 0 vs. SH II	1.1 (0.9 to 1.3)	0.08	0.21
SH 0 vs. SH III	1.2 (0.9 to 1.4)	0.10	0.21
SH 0 vs. SH IV	1.0 (0.7 to 1.3)	0.92	0.92
SH 0-I vs. SH II-IV	1.7 (1.0 to 2.8)	0.04*	

*aOR* adjusted odds ratio, Adjusted *P* value with Benjamini-Hochberg.

* Statistical significance, *P* < 0.05 and values are marked in bold. ** Statistical significance, *P* < 0.001 and values are marked in bold

### Predictors of hyperdensities in the subarachnoid space

Two variables were potentially strongly associated with SH on FDCT, namely the location of vessel occlusion distally in M2 and the number of device passes (Table 3).

**Table 3:** Predictors of subarachnoid hyperdensities

	Odds ratio	95% CI	*P*-value
Age	0.99	0.97 to 1.00	0.15
Sex	1.37	0.89 to 2.10	0.15
NIHSS on admission	1.01	0.98 to 1.04	0.49
Intravenous thrombolysis	1.01	0.65 to 1.58	0.95
Number of passes	1.36	1.19 to 1.56	< 0.001**
Anticoagulation	0.64	0.34 to 1.21	0.16
Antiplatelet	1.12	0.69 to 1.82	0.63
Occlusion site (MVO)	2.62	1.65 to 4.18	< 0.001**
Occlusion site (SVO)	2.46	0.89 to 6.82	0.08

* Statistical significance, *P* < 0.05 and values are marked in bold. ** Statistical significance, *P* < 0.001 and values are marked in bold

Multivariable logistic regression analysis demonstrated increased odds of developing SH on FDCT in patients with MVOs (OR = 2.62; 95% CI, 1.65–4.18) and in patients requiring more device passes (maneuvers) (OR = 1.36; 95% CI, 1.19–1.56).

Logistic regression was performed on subtypes of M2 occlusions and device passes, in which the reference was set to LVO and number of passes (1–3). The analysis indicated that patients with co- and non-dominant M2 occlusions had the highest odds of presenting SH in categories I–IV (OR = 3.78; 95% CI, 2.18–6.57), followed by number of thrombectomy maneuvers > 6 (OR = 3.45; 95% CI, 1.17–10.16), then numbers of maneuvers between 4 and 6 (OR = 2.75; 95% CI, 1.56–4.84), and dominant M2 occlusions (OR = 3.07; 95% CI, 1.68–5.59) (Table 4).

**Table 4:** Logistic regression analysis on subtypes of M2 occlusions and device passes

	OR	95%-CI
Number of passes 4–6	2.75	1.56 to 4.84
Number of passes > 6	3.45	1.17 to 10.16
M2 dominant	3.07	1.68 to 5.59
M2 non-or co-dominant	3.78	2.18 to 6.57

*OR* odds ratio

## Discussion

Our study highlights the relationship between SH detected on post-interventional FDCT and certain clinical and procedural factors. We found post-interventional SH in 41.6% of the patients after MT for anterior circulation occlusions. They were most frequent in patients with dominant M2 occlusions (53.9%) and those with co- or non-dominant M2 occlusions (63%). This finding does not align with early data from registries, which reported lower rates of post-interventional SH [1, 3]. However, distal occlusions were either not included or were underrepresented in both of these studies.

Our analysis demonstrated that increasing severity of SH correlates with worse outcomes. Specifically, SH grades II–IV were associated with higher odds for early neurologic deterioration, poorer functional outcomes, and increased mortality. This is consistent with previous studies, which also noted that severe SH is indicative of negative clinical trajectories and higher mortality [3]. Patients with SH grade I experienced outcomes similar to those without SH, suggesting that small amounts of SH might have a less adverse effect on prognosis. Detection of SH may warrant immediate therapeutic action by the interventionist e.g. stricter blood pressure management and discontinuation or adaptation of dual-antiplatelet therapy.

Follow-up imaging within 24 h revealed that, in a substantial proportion of cases, SH, particularly the lower-grade ones, had resolved or diminished (27.73% vs. 41.6%). However, FDCT cannot distinguish between contrast media leakage and true hemorrhage. Follow-up dual energy CT helps in distinguishing between blood and contrast leakage [18].

This investigation showed that several procedural factors were linked to an increased risk of SH. These include multiple thrombectomy attempts and distal occlusions, especially in smaller diameter M2 branches.

The highest odds of presenting SH were seen in patients with co- and non-dominant M2 occlusions (OR = 3.78; 95% CI, 2.18 to 6.57). This finding could be attributed to mechanical injury to the endothelium caused by stent retrievers, microcatheters, or guidewires. Navigating such instruments in smaller, more tortuous vessels causes greater wall shear stress, which may result in vessel wall injury. The radial force exerted on the vessel wall and the traction force applied to retrieve a device have been reported to be higher in smaller and distally located vessels [19]. This may also cause rupture of nearby arterioles and venules.

There was no significant difference in the reperfusion rates among the different caliber M2-occlusions (*P* = 0.91). However, this represents a dilemma: although it is technically possible to recanalize these occlusions, the risk of peri-interventional complications is significantly higher.

A larger number of thrombectomy maneuvers (> 6) was also associated with a higher OR of developing SH (OR = 3.45; 95% CI, 1.17–10.16), presumably due to repeated vessel wall injury.

The diameters in MVOs are smaller than the LVOs and the inflected brain ischemia should therefore be smaller; in theory, this should result in less severe symptoms. However, this is not the case, due to the broad variation of M2 branching [13] and consequently the arterial territory at risk. Smaller SRs with lower radial force, as well as smaller diameter and more trackable aspiration catheters are available on the market to facilitate MT in MVOs and to help achieve high recanalization rates. Nevertheless, it remains unclear whether the benefits outweigh the increased likelihood of complications during MT of patients with MVO. The recently published ESCAPE-MeVO and DISTAL trials delivered surprising results and demonstrated that MT of MVO didn’t lead to better outcomes compared to conservative care.

Additionally, the trials primarily reported on symptomatic intracranial hemorrhage (sICH) as a safety outcome. While sICH encompasses various types of hemorrhagic events, the trials did not provide specific data on the incidence or grading of SAH post-thrombectomy. They didn’t report higher rates of ICH [20, 21]. In the DISTAL trail the MVOs are subcategorized into distal and proximal occlusions. There is currently no available data on the sub-analysis of the caliber-dominance and associated complications in both trials.

Periprocedural SHs are sequelae of MT and thus it is worthwhile–in the light of our results and especially those in patients with MVO occlusions– to consider which technique may be most vessel-protective. In that regard, the direct aspiration first-pass technique (ADAPT) is reported to be associated with lower distal embolization rates and less thrombus fragmentation [22]. In an experimental study, direct aspiration and the combined technique were tested in a vascular model. The combined technique appeared to be more harmful, but direct aspiration had a lower rate of first-pass recanalization [23].

The first-line technique in both the tertiary centers participating in this investigation was the combined technique (stent retriever and distal aspiration). Only seven of the patients included in this investigation were treated with an aspiration catheter alone. Two of them presented with a mild SH (grade I). Thus, a direct comparison of the two techniques was not possible.

Subgroup analysis of larger studies estimates that MVOs account for 25–40% of cases of ischemic stroke [24] and intravenous thrombolytics have been shown to result in recanalization of about one-half of MVOs [25, 26] when used without MT. This means that many patients could still benefit from MT. The reported high rates of SH, together with the associated clinical deterioration and higher mortality, underscore the need for careful selection for MT of patients with MVOs to maximize benefits and minimize potential harm. In the current investigation, we did not include the time from onset-to-revascularization in the analysis. Due to a high proportion of external hospital referrals, accurate and complete documentation of symptom onset was often unavailable.

In conclusion, SH is a common and important finding after MT, particularly in distal and smaller diameter vessels. The implications of SH are substantial, and a better understanding of the underlying mechanisms, including vessel damage and disruption of the blood–brain barrier, may aid in the development of safer devices as well as the implementation of more effective recanalization techniques, ultimately improving patient outcomes.

## Limitations

The investigation has several limitations. Firstly, its retrospective study design carries inherent biases. Secondly, unreported interventional technical aspects of the underlying procedures, use of different catheters and SRs and heterogenous peri- and post-interventional medication regimes are possible.

In addition, several factors were not taken into account (most distal site of microcatheter, diameter and length of different SR, etc.).

## Electronic supplementary material

Below is the link to the electronic supplementary material.

Supplementary Material 1

## Data Availability

Individual participant data that underlie the results reported in this article (after deidentification) will be available upon reasonable request. These proposals should be directed to the corresponding author.
