# Supplementary material for: A bicentric study on the prevalence and clinical relevance of subarachnoid hyperdensities on flat-detector CT after thrombectomy in dominant, co-dominant, and non-dominant M2 occlusions
Source: Neuroradiology. 2025 Jun 19;67(8):2155–65. doi: 10.1007/s00234-025-03679-x (PMC12494646; doi:10.1007/s00234-025-03679-x)
Supplement: Supplementary file 1 — Supplementary Material 1 [file 234_2025_3679_MOESM1_ESM.docx]

**Supplemental material 1**

Procedural characteristics stratified by subarachnoid hyperdensities, device passes subcategories and different caliber M2 occlusions.

| Group | SH 0 | SH I | SH II | SH III | SH IV | p |
| --- | --- | --- | --- | --- | --- | --- |
| ***Median (lq-uq), [N] , % (n/N)*** | *240* | *77* | *54* | *28* | *12* |  |
| Baseline intracranial occlusion site    LVO | 66% (158) | 42% (32) | 46% (25) | 39% (11) | 67% (8) | **0.001*** |
| MVO | 30% (73) | 55% (42) | 46% (25) | 54% (15) | 33% (4) |  |
| SVO | 3.8% (9) | 3.9% (3) | 7.4% (4) | 7.1% (2) |  |  |
| Number of device passes |  |  |  |  |  |  |
|  | 1 [1.0- 2.0] | 2 [1.0- 4.0] | 2.5 [1.2- 3.8] | 3 [2.0- 4.0] | 2 [1.0- 4.0] | **<0.001**** |
| Device passes categories: |  |  |  |  |  |  |
| 1-3 | 86% (206) | 69% (53) | 74% (40) | 61% (17) | 67% (8) | **0.004*** |
| 4-6 | 11% (26) | 21% (16) | 22% (12) | 29% (8) | 33% (4) |  |
| >6 | 2.5% (6) | 9.1% (7) | 3.7% (2) | 3.6% (1) | 0% (0) |  |
|  |  |  |  |  |  |  |
| M2 occlusions: | 29% (70) | 56% (43) | 52% (28) | 50% (14) | 33% (4) | <**0.001**** |
| Dominant M2 | 9.6% (23) | 17% (13) | 22% (12) | 21% (6) | 25% (3) |  |
| Co-Dominant M2 | 7.1% (17) | 26% (20) | 22% (12) | 25% (7) | 0% (0) |  |
| Non-Dominant M2 | 4.2% (10) | 7.8% (6) | 3.7% (2) | 3.6% (1) | 0% (0) |  |

LVO; large vessel occlusion, MVO; medium vessel occlusion, SVO; small vessel occlusion.

* Statistical significance, P < 0.05 and values are marked in bold. ** Statistical significance, P < 0.001 and values are marked in bold.
